# Outcomes by Class of Anticoagulant Use for Nonvalvular Atrial Fibrillation in Patients With Active Cancer

**DOI:** 10.1016/j.jaccao.2022.07.004

**Published:** 2022-09-20

**Authors:** Adam S. Potter, Ashley Patel, Muzamil Khawaja, Christopher Chen, Henry Zheng, Jessica Kaczmarek, Feng Gao, Kaveh Karimzad, Juhee Song, Efstratios Koutroumpakis, Shaden Khalaf, Cezar Iliescu, Anita Deswal, Nicolas L. Palaskas

**Affiliations:** a Department of Cardiology, University of Texas MD Anderson Cancer Center, Houston, Texas, USA; b Department of Internal Medicine, Baylor College of Medicine, Houston, Texas, USA; c Department of Biostatistics, University of Texas MD Anderson Cancer Center, Houston, Texas, USA

**Keywords:** bleeding, cancer, DOAC, nonvalvular atrial fibrillation, stroke, vitamin K antagonist, warfarin, CVA, cerebrovascular accident, DOAC, direct oral anticoagulant agent, GIB, gastrointestinal bleeding, ICH, intracranial hemorrhage, LMWH, low–molecular weight heparin, NVAF, nonvalvular atrial fibrillation, TIA, transient ischemic attack, VKA, vitamin K antagonist, VTE, venous thromboembolism

## Abstract

**Background:**

The choice of anticoagulant agent for patients with nonvalvular atrial fibrillation (NVAF) in the setting of active cancer has not been well studied.

**Objectives:**

The aim of this study was to compare the rates of cerebrovascular accident (CVA), gastrointestinal bleeding (GIB), and intracranial hemorrhage (ICH) in patients treated with direct oral anticoagulant agents (DOACs) compared with warfarin for NVAF in patients with active cancer.

**Methods:**

This was a retrospective electronic medical record review of eligible patients treated at a cancer hospital. The outcome events were CVA; GIB; ICH; the composite of GIB, CVA, or ICH; and overall mortality. Propensity score matching (1:1) was conducted to select comparable patients receiving warfarin vs DOACs. Fine-Gray models were fitted for each outcome event.

**Results:**

The study cohort included 1,133 patients (mean age 72 ± 8.8 years, 42% women), of whom 74% received DOACs (57% received apixaban). After propensity score matching, 195 patients were included in each anticoagulant agent group. When comparing warfarin with DOACs, there were similar risks for CVA (subdistribution HR: 0.738; 95% CI: 0.334-1.629); ICH (subdistribution HR: 0.295; 95% CI: 0.032-2.709); GIB (subdistribution HR: 1.819; 95% CI: 0.774-4.277); and the composite of GIB, CVA, or ICH (subdistribution HR: 1.151; 95% CI: 0.645-2.054).

**Conclusions:**

Patients with active cancer had similar risks for CVA, ICH, and GIB when treated with DOACs compared with warfarin for NVAF.

The choice of anticoagulant agent for patients with non-valvular atrial fibrillation (NVAF) has shifted from primarily vitamin K antagonists, such as warfarin, to direct oral anticoagulant agents (DOACs), including apixaban, rivaroxaban, dabigatran, and edoxaban. Several pivotal randomized controlled trials comparing DOACs with warfarin showing similar efficacy led to their approval. With ease of use and no requirement for blood level monitoring, DOACs have become the predominant anticoagulant agents of choice for patients with NVAF. However, there are limited data regarding the use of DOACs for NVAF in patients with active cancer. A post hoc analysis of the randomized controlled trials (ENGAGE AF–TIMI 48 [Effective Anticoagulation With Factor Xa Next Generation in Atrial Fibrillation–Thrombolysis In Myocardial Infarction Study 48], ROCKET-AF [Rivaroxaban Once Daily Oral Direct Factor Xa Inhibition Compared With Vitamin K Antagonism for Prevention of Stroke and Embolism Trial in Atrial Fibrillation], and ARISTOTLE [Apixaban for Reduction in Stroke and Other Thromboembolic Events in Atrial Fibrillation])^1^, ^2^, ^3^, ^4^ comparing DOACs with warfarin have a minority of patients with any history of cancer and even fewer patients with active cancer. Observational studies have been limited to large database claims data, not allowing assessment of individual patient embolic and bleeding risks or of adjudication of adverse ischemic and hemorrhagic events.^5^^,^^6^ Cancer is associated with both prothrombotic and increased bleeding risks, and the risks and benefits of anticoagulation must be weighed. Although data are limited on the preferred oral anticoagulant agent in patients with NVAF and active cancer, there have been previous randomized controlled trials and a recent meta-analysis that compared DOACs and low–molecular weight heparin (LMWH) in treating venous thromboembolism (VTE) in patients with active cancer.^7^, ^8^, ^9^, ^10^, ^11^ These studies showed that DOACs were either noninferior or superior to LMWH in preventing recurrent VTE, with generally no increased incidence of major bleeding, although in certain studies clinically relevant nonmajor bleeding was increased compared with LMWH, primarily in patients with gastrointestinal malignancies. These data overall suggest that DOACs are safe in patients with cancer for the prevention of recurrent VTE but do not directly address their use in patients with NVAF. Current guidelines do not indicate which anticoagulant agent to choose for patients with vs without malignancy^12^; furthermore, embolic and bleeding risk scores do not include the presence of active cancer or history of cancer. In this study, we aimed to assess the embolic and bleeding risk of patients with NVAF and active cancer and to compare ischemic and hemorrhagic events between patients receiving warfarin vs DOACs using adjudicated individual medical record review.

## Methods

### Study population

This retrospective cohort study included all patients with active cancer and NVAF at the University of Texas MD Anderson Cancer Center between June 1, 2013, and December 31, 2018. The electronic medical record was queried for the diagnosis of atrial fibrillation between these dates and for the presence of any of the following medications at any time: apixaban, rivaroxaban, dabigatran, edoxaban, and warfarin. Manual chart review was then performed to obtain clinical data. Active cancer at the time of anticoagulation initiation was defined as the presence of disease or still receiving cancer therapeutic agents. Patients with valvular atrial fibrillation as defined in the American Heart Association/American College of Cardiology guidelines were excluded, and only those patients prescribed anticoagulation for atrial fibrillation thromboembolic risk mitigation were included.^13^ The time of anticoagulation initiation was calculated as the earliest that first-time anticoagulation was prescribed at our center or if patients presented to the University of Texas MD Anderson Cancer Center with histories of atrial fibrillation already on anticoagulation, in which case the date of initiation was defined as the first presentation to the University of Texas MD Anderson Cancer Center. CHA_2_DS_2_-VASc^14^ and HAS-BLED^15^ scores at time of anticoagulation initiation were calculated on the basis of available clinical data. Outcomes of stroke, including transient ischemic attack (referred to collectively as cerebrovascular accident [CVA]), and intracranial hemorrhage (ICH) were defined by imaging and/or neurology consultation notes confirming the events. Outcomes of gastrointestinal bleeding (GIB) were defined by endoscopic evidence of GIB and/or gastroenterology notes documenting GIB. When events were identified on chart review, a second reviewer reviewed the charts to confirm the conclusions. Uncontrolled hypertension was defined as systolic blood pressure > 160 mm Hg, and alcohol use was defined as ≥8 drinks per week, consistent with the HAS-BLED criteria. Patients who were switched between different anticoagulant agents before an outcome or last follow-up occurred were excluded. The Institutional Review Board approved the protocol, and the requirement to obtain informed consent was waived because of the retrospective nature of the study. The study protocol conformed to the ethical guidelines of the 1975 Declaration of Helsinki, as reflected in a priori approval by the institution’s human research committee.

### Statistical analysis

Patient characteristics are expressed as mean ± SD or median (IQR) for continuous variables and frequency (percentage) for categorical variables. Patient characteristics were compared between DOAC and warfarin groups using 2-sample Student’s *t*-test or the Wilcoxon rank sum test for continuous variables and the chi-square or Fisher exact test for categorical variables, as appropriate. The Kaplan-Meier method was used to estimate the time free from outcome events (GIB, ICH, and CVA) and overall survival. The time to first event (GIB, ICH, and CVA) was calculated as the time from anticoagulation initiation to first individual event and when combining all outcomes was defined as the time from anticoagulation initiation to first of any of the 3 main outcome events (GIB, ICH, and CVA). Overall survival time was defined as the time interval from initiation of treatment to death. Censoring was performed if the patient was alive at last follow-up and at death if this occurred before the event of interest. Univariable Fine-Gray models, considering death as a competing risk, were used to compare CVA-free survival, GIB-free survival, ICH-free survival, event-free survival, and overall survival between warfarin and DOACs in propensity score–matched cohorts. The details of selection of propensity-matched cohorts are described in the section on propensity score calculation. Cox proportional hazards models for CVA, GIB, and any of the adverse events were fitted as a sensitivity analysis. Additional analysis taking into account intraclass correlation was performed using clustered Fine-Gray models and clustered Cox regression models, which showed similar results to the primary analysis (data not shown). Results are presented as HRs with 95% CIs. Because of the differences in time when patients received either DOACs or warfarin, we artificially censored at 5 years. A *P* value <0.05 indicated statistical significance. SAS version 9.4 (SAS Institute) was used for data analysis.

### Propensity score calculation

Propensity score matching (1:1) without replacement on the basis of year of anticoagulation initiation, gastrointestinal malignancy, and individual parameters of the CHA_2_DS_2_-VASc and HAS-BLED scores was performed to select comparable patients receiving warfarin vs DOACs. The final multivariable logistic regression model to predict anticoagulation included individual variables of the CHA_2_DS_2_-VASc^14^ and HAS-BLED^15^ scores as defined by the referenced studies (age, gender, heart failure, hypertension, diabetes mellitus, CVA, vascular disease, prior major bleeding, alcohol use, uncontrolled hypertension, renal disease, and medication predisposing to bleeding) in addition to the presence of gastrointestinal malignancy, year that anticoagulation was initiated (1995-2010 vs 2011-2015 vs 2016-2020), and overall HAS-BLED score. Two individual variables within the overall HAS-BLED score, labile international normalized ratio and abnormal liver function, could not be matched for, given their association with the use of DOACs compared with warfarin. The overall HAS-BLED score was added to the matching variables because after matching for individual variables within the scores, there were still significant differences in HAS-BLED score between the DOAC and warfarin groups. A multivariable logistic regression model was used in predicting receipt of warfarin (Supplemental Table 1). The caliper width used was 0.2. The propensity score was obtained for each patient using this model, and 1:1 propensity score–matched cohorts were selected using the OnetoManyMTCH macro, using a greedy algorithm.^16^

## Results

**Figure 1:**
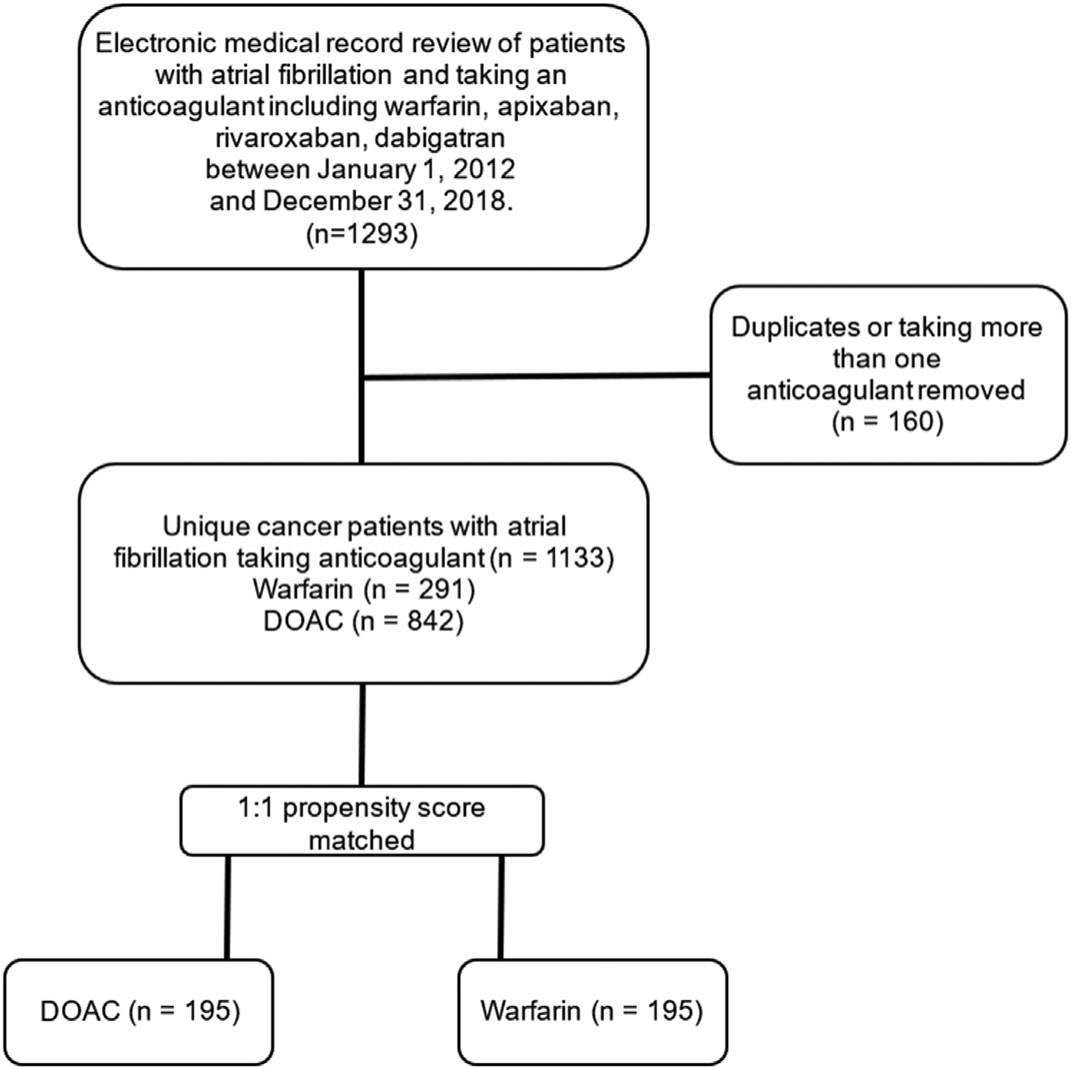
Study Flow Diagram Criteria used for the selection of patients. The inclusion and exclusion criteria of this study yielded 1,293 patients with active cancer and nonvalvular atrial fibrillation taking oral anticoagulant agents. Patients taking more than 1 anticoagulant agent, those who had changes in anticoagulant agents, those with diagnoses of valvular atrial fibrillation, and duplicate patients were removed. The remaining 1,133 patient were 1:1 propensity matched into direct oral anticoagulant agent (DOAC) and warfarin cohorts.

Initial query of the medical record returned 1,213 patients. After applying selection criteria, 1,133 patients with active cancer and NVAF receiving anticoagulation were identified (Figure 1). Of these patients, 842 (74.3%) were prescribed DOACs and 291 (25.7%) were prescribed warfarin. Of the patients prescribed DOACs, 482 (57.2%) were prescribed apixaban, 303 (35.9%) were prescribed rivaroxaban, 54 (6.4%) were prescribed dabigatran, and 3 (0.4%) were prescribed edoxaban. Patients on DOACs were older (mean age 73 ± 8.6 years) compared with warfarin users (mean age 70 ± 9.0 years) (*P* < 0.001). In the whole cohort, the baseline risk scores for both thromboembolism and bleeding were higher for those on warfarin compared with DOACs (CHA_2_DS_2_-VASc score 3.5 ± 1.6 vs 3.3 ± 1.7 [*P* = 0.040], HAS-BLED 2.0 ± 1.1 vs 1.8 ± 1.0 [*P* = 0.001]) (Table 1). Approximately 42% were women, and the most common malignancies were hematologic (21%), genitourinary (20%), breast (15%), and gastrointestinal (12%). Aggregate numbers of types of cancer for patients listed in the “other” cancer category are listed in Supplemental Table 2. Differences in baseline characteristics on the basis of sex are provided in Supplemental Table 3. When we artificially censored at 5 years, and before propensity score matching, 145 (20.5%) died, 59 (8.4%) had CVAs, 6 (1.1%) had ICH, 59 (8.3%) had GIB, and 114 (15.6%) had any adverse event, which included a composite of stroke, ICH, or GIB, with percentages reported as Fine-Gray cumulative incidence estimates. The median follow-up time when we artificially censored at 5 years, estimated using the reverse Kaplan-Meier method, was 943 days (95% CI: 906-1,008 days), and the median overall survival was not reached.

**Table 1:** Baseline Characteristics of Patients Taking DOACs or Warfarin in the Overall Cohort Prior to Matching

	DOAC (n = 842)	Warfarin (n = 291)	*P* Value	Standardized Mean Difference
Sex			0.009	0.180
Male	473 (56)	189 (65)		
Female	369 (44)	102 (35)		
Age, y	73.3 ± 8.6	70.1 ± 9.0	<0.001	−0.364
Race			0.34	−0.048
Black	47 (6)	23 (8)		
White	748 (89)	254 (87)		
Other	47 (6)	14 (5)		
CHA_2_DS_2_-VASc score	3.3 ± 1.7	3.5 ± 1.6	0.040	0.142
HAS-BLED score	1.8 ± 1.0	2.0 ± 1.1	0.001	0.221
Year anticoagulation started			<0.001	0.516
1995-2010	25 (3)	101 (35)		
2011-2015	193 (23)	136 (47)		
2016-2020	624 (74)	54 (19)		
Comorbidities				
Heart failure	118 (14)	72 (25)	<0.001	−0.274
Hypertension	646 (77)	249 (86)	0.001	−0.228
Uncontrolled hypertension	130 (15)	27 (9)	0.009	0.188
Diabetes	192 (23)	70 (24)	0.66	−0.030
CVA	128 (15)	44 (15)	0.97	0.002
Vascular disease	223 (27)	103 (35)	0.004	−0.194
Prior major bleeding	66 (8)	17 (6)	0.26	0.079
Hyperlipidemia	721 (86)	238 (82)	0.12	−0.104
Alcohol use	22 (3)	9 (3)	0.67	−0.029
Renal disease	44 (5)	18 (6)	0.54	−0.041
Labile INR	2 (0.2)	108 (37)	<0.001	0.137
Liver disease	27 (3)	0 (0)	0.002	—
Medication predisposing to bleeding	269 (32)	75 (26)	0.048	—
Cancer type			0.18	—
Breast	136 (16)	36 (12)		
Genitourinary	153 (18)	68 (23)		
Gastrointestinal	99 (12)	38 (13)	0.49^a^	−0.047^b^
Hematologic^c^	177 (21)	59 (20)		
Lung	63 (8)	29 (10)		
Skin^d^	88 (11)	24 (8)		
Other^e^	126 (15)	37 (13)		

Values are n (%) or mean ± SD.

CVA = cerebrovascular accident; DOAC = direct oral anticoagulant agent; INR = international normalized ratio.

a *P* value comparing gastrointestinal vs nongastrointestinal cancer.

b Mean standardized difference of gastrointestinal cancer type to nongastrointestinal cancer type.

c Acute myeloid leukemia, acute lymphocytic leukemia, chronic lymphocytic leukemia, chronic myeloid leukemia, diffuse large B cell lymphoma, multiple myeloma, myelofibrosis, monoclonal gammopathy of unknown significance, polycythemia vera, essential thrombocythemia, follicular lymphoma, hairy cell, Hodgkin lymphoma, marginal zone lymphoma.

d Squamous cell carcinoma, melanoma, basal cell carcinoma, mycosis fungoides, dermatofibroma, Merkel cell.

e Sarcoma, gynecologic, central nervous system, bone, head and neck.

Patients were 1:1 propensity matched on the basis of variables presented in the “Methods” section, resulting in 195 patients who received DOACs and 195 who received warfarin. The median follow-up time, using the reverse Kaplan-Meier method and artificially censored at 5 years, was 1,500 days (95% CI: 1,407-1,618 days), and the median overall survival was not reached. The median follow-up time was similar between those receiving DOACs and warfarin (1,407 days [95% CI: 1,255-1,559 days] and 1,618 days [95% CI: 1,471-1,702 days], respectively). Baseline patient characteristics are summarized by treatment group (Table 2). After matching, baseline characteristics were balanced between the DOAC and warfarin groups; Tables 1 and 2 show mean standardized differences before and after matching. In addition, a detailed description of differences between unmatched and matched cohorts is presented in Supplemental Table 4.

**Table 2:** Baseline Characteristics of Patients Taking DOACs or Warfarin in the 1:1 Propensity Score–Matched Cohorts

	DOAC (n = 195)	Warfarin (n = 195)	*P* Value	Standardized Mean Difference	Variance Ratio
Sex			0.78	0.032	0.986
Male	118 (61)	121 (62)			
Female	77 (40)	74 (38)			
Age, y	72.5 ± 8.4	71.6 ± 9.1	0.26	−0.105	1.181
Race			0.34	−0.048	
Black	7 (4)	14 (7)	0.29	−0.104	1.329
White	179 (92)	173 (89)			
Other	9 (5)	8 (4)			
CHA_2_DS_2_-VASc score	3.5 ± 1.8	3.5 ± 1.6	0.67	−0.033	0.812
HAS-BLED score	1.8 ± 1.0	2.0 ± 1.1	0.001	0.221	1.226
Year anticoagulation started			0.84	−0.021	1.007
1995-2010	24 (12)	28 (14)			
2011-2015	115 (59)	113 (58)			
2016-2020	56 (29)	54 (28)			
Comorbidities					
Heart failure	44 (23)	44 (23)	1.00	0	1.000
Hypertension	160 (82)	166 (85)	0.41	−0.083	0.860
Uncontrolled hypertension	22 (11)	24 (12)	0.75	−0.032	1.078
Diabetes	49 (25)	50 (26)	0.91	−0.012	1.013
CVA	33 (17)	33 (17)	1.00	0	1.000
Vascular disease	59 (30)	64 (33)	0.59	−0.055	1.045
Prior major bleeding	14 (7)	14 (7)	1.00	0	1.000
Hyperlipidemia	163 (84)	164 (84)	0.89	0.014	0.974
Alcohol use	3 (2)	4 (2)	1.00	−0.039	1.326
Renal disease	12 (6)	12 (6)	1.00	0	1.000
Labile INR	1 (0.5)	44 (23)	<0.001	—	—
Liver disease	10 (5)	0 (0)	0.002	—	—
Medication predisposing to bleeding	47 (24)	51 (26)	0.64	−0.047	1.056
Cancer type			0.68	—	—
Breast	32 (16)	25 (13)			
Genitourinary	40 (21)	46 (24)			
Gastrointestinal	29 (15)	24 (12)	0.46^a^	0.075^b^	0.853
Hematologic^c^	36 (18)	36 (18)			
Lung	11 (6)	18 (9)			
Skin^d^	23 (12)	19 (10)			
Other^e^	24 (12)	27 (14)			

Values are n (%) or mean ± SD.

Abbreviations as in Table 1.

a *P* value comparing gastrointestinal vs nongastrointestinal cancer.

b Mean standardized difference of gastrointestinal cancer type to nongastrointestinal cancer type.

c Acute myeloid leukemia, acute lymphocytic leukemia, chronic lymphocytic leukemia, chronic myeloid leukemia, diffuse large B cell lymphoma, multiple myeloma, myelofibrosis, monoclonal gammopathy of unknown significance, polycythemia vera, essential thrombocythemia, follicular lymphoma, hairy cell, Hodgkin lymphoma, marginal zone lymphoma.

d Squamous cell carcinoma, melanoma, basal cell carcinoma, mycosis fungoides, dermatofibroma, Merkel cell.

e Sarcoma, gynecologic, central nervous system, bone, head and neck.

### Outcomes in patients on DOACs vs warfarin

**Figure 2:**
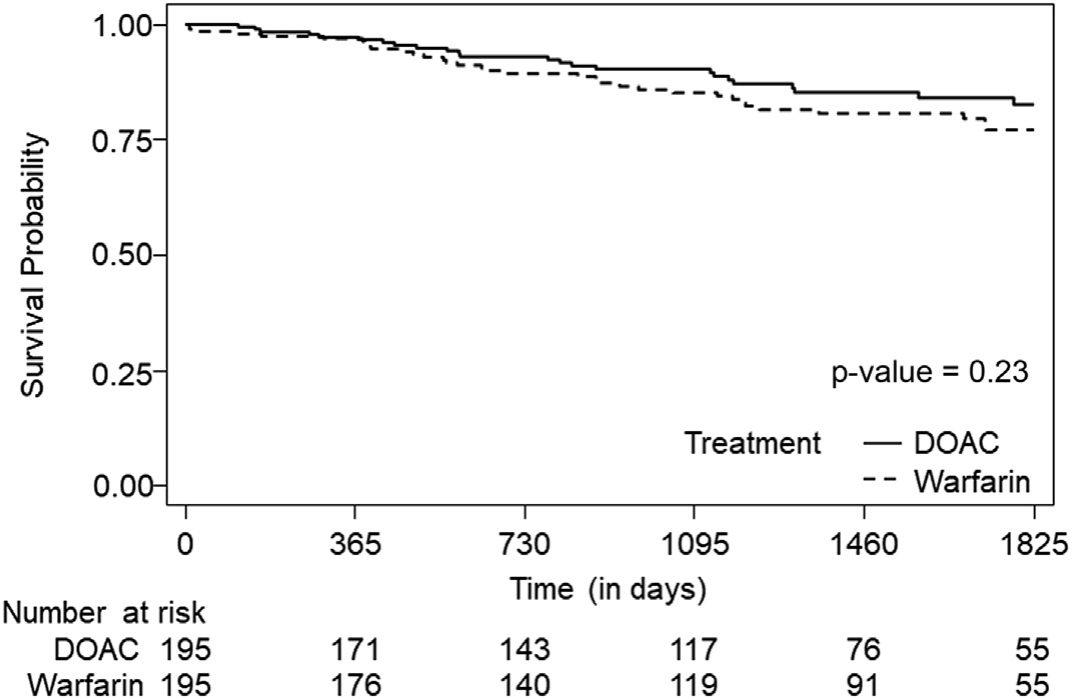
Kaplan-Meier Plot for Overall Survival Comparison of overall survival in the propensity-matched cohort of patients receiving direct oral anticoagulant agents (DOACs) vs warfarin. Time-to-event comparison using a Kaplan-Meier plot was used, and survival curves were compared using a log-rank test. No significant difference was observed in overall survival between patients receiving DOACs and those receiving warfarin (*P* = 0.23). This analysis demonstrated that anticoagulant agent type was not significantly associated with overall survival.

**Figure 3:**
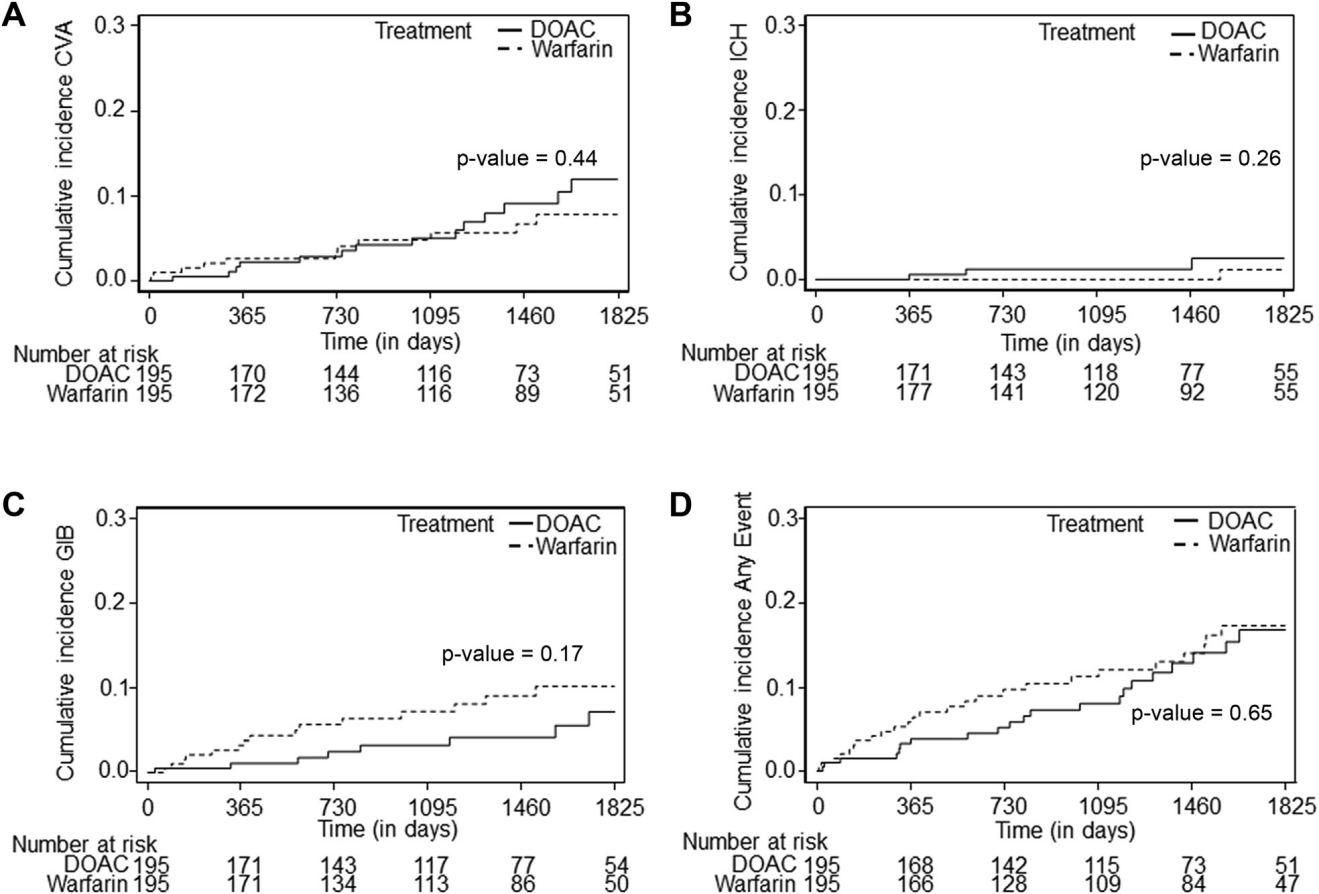
Cumulative Incidence Plots for Outcomes by Anticoagulant Agent Comparison of cumulative incidence of cerebrovascular accident (CVA) **(A)**, intracranial hemorrhage (ICH) **(B)**, gastrointestinal bleeding (GIB) **(C)**, and any event **(D)** in the propensity-matched cohort of patients receiving direct oral anticoagulant agents (DOACs) vs warfarin. The Aalen-Johansen method was used. This analysis demonstrated that anticoagulant agent type was not significantly associated with CVA, ICH, GIB, or any event.

In the propensity-matched cohort, 58 patients (20.0%) died, 23 (7.8%) had GIB, 25 (8.8%) had CVAs, 4 (1.6%) had ICH, and 46 (15.5%) had at least 1 adverse event, with percentages reported as Fine-Gray cumulative incidence estimates. Kaplan-Meier and cumulative incidence plots showed no significant differences in overall survival, CVA-free survival, ICH-free survival, GIB-free survival, or any event-free survival (Figures 2 and 3). Time-to-event analysis using death as a competing risk demonstrated no significant differences in CVA-free survival, ICH-free survival, GIB-free survival, or any event-free survival between the DOAC and warfarin groups (Table 3). Fine-Gray models demonstrated similar risk for CVA and ICH between the warfarin and DOAC groups that was not significantly different (subdistribution HRs: 0.738 [95% CI: 0.334-1.629; *P* = 0.45] and 0.295 [95% CI: 0.032-2.709; *P* = 0.28], respectively) (Table 3). Similarly, Fine-Gray models demonstrated no differences in GIB and the composite event between the warfarin and DOAC groups (subdistribution HRs: 1.819 [95% CI: 0.774-4.277; *P* = 0.17] and 1.151 [95% CI: 0.645-2.054; *P* = 0.63], respectively) (Table 3). The results based on Cox regression models were comparable with Fine-Gray models for time to CVA, ICH, GIB, and the composite event (Supplemental Table 5).

**Table 3:** Fine-Gray Models for CVA, ICH, GIB, and Composite Event Using 1:1 Propensity Score–Matched Cohorts

Covariate	Fine-Gray Models							
	CVA		ICH		GIB		Composite Event (CVA, ICH, and/or GIB)	
	aHR (95% CI)	*P* Value	aHR (95% CI)	*P* Value	aHR (95% CI)	*P* Value	aHR (95% CI)	*P* Value
Type of anticoagulant								
DOAC	1.000	—	1.000		1.000		1.000	
Warfarin	0.738 (0.334-1.629)	0.45	0.295 (0.032-2.709)	0.28	1.819 (0.774-4.277)	0.17	1.151 (0.645-2.054)	0.63

aHR = adjusted subdistribution HR; GIB = gastrointestinal bleed; ICH = intracranial hemorrhage; other abbreviations as in Table 1.

## Discussion

To our knowledge, we report one of the first real-world studies using clinical data in patients with active cancer and NVAF, comparing adjudicated outcomes between patients on DOACs and those on warfarin. We observed that there were no significant differences in CVA, ICH, and GIB in patients on DOACs compared with those on warfarin (Central Illustration).

**Central Illustration:**
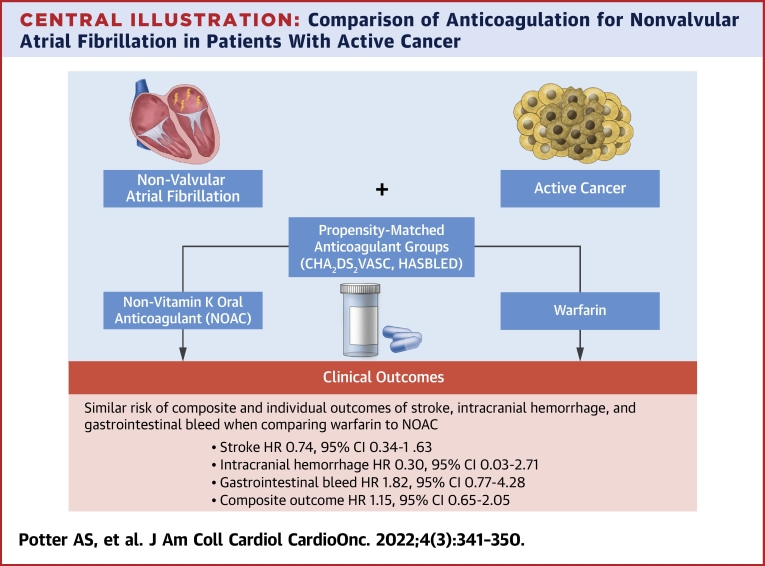
Comparison of Anticoagulation for Nonvalvular Atrial Fibrillation in Patients With Active Cancer Overall results comparing outcomes of cerebrovascular accident (CVA), intracranial hemorrhage (ICH), and gastrointestinal bleeding (GIB) in patients with active cancer and nonvalvular atrial fibrillation (NVAF) receiving warfarin compared with direct oral anticoagulant agents (DOACs) in a single-center retrospective cohort study using propensity-matched cohorts compared with Fine-Gray models. There was similar risk for CVA (subdistribution HR: 0.74; 95% CI: 0.34-1.63), ICH (subdistribution HR: 0.30; 95% CI: 0.03-2.71), GIB (subdistribution HR: 1.82; 95% CI: 0.77-4.28), and the composite event of CVA, ICH, or GIB (subdistribution HR: 1.15; 95% CI: 0.65-2.05) when comparing patients receiving warfarin and those receiving DOACs. Given the similar efficacy and adverse event profile of DOACs and warfarin for NVAF, they should be considered for use in patients with active cancer.

Cancer is known to cause both a prothrombotic state and an increased risk for bleeding, which makes its particularly challenging to treat this patient population with optimal anticoagulation in the setting of NVAF.^17^, ^18^, ^19^, ^20^ Previous landmark clinical trials have established the efficacy and safety of DOACs in the general population. The trials demonstrated that DOACs are at least as effective in preventing stroke in patients with NVAF compared with warfarin, leading to recommendations for their use as first-line anticoagulation agents for NVAF in recent guidelines.^12^^,^^21^, ^22^, ^23^, ^24^ However, patients with active malignancies are underrepresented in these trials; therefore our findings have important clinical significance to help guide management of anticoagulation in cancer patients with NVAF.

The results of our real-world study add to existing subgroup analyses from randomized clinical trials. Post hoc analysis of the ENGAGE AF–TIMI 48 trial, which randomized patients with NVAF to edoxaban vs warfarin, identified 1,153 patients postrandomization with new malignancies, defined as new cancer or recurrence of remote cancer. Similar to the results of our study, there was no significant difference in the incidence of stroke or systemic embolism (HR: 0.60; 95% CI: 0.31-1.15) and major bleeding (HR: 0.98; 95% CI: 0.69-1.40) in patients receiving edoxaban vs warfarin.^1^ Post hoc analysis of the ROCKET AF trial revealed that patients with malignancies, defined as a history of having cancer, included 640 patients of whom only 50 had active cancer, and did not show significant differences in stroke (HR: 0.52; 95% CI: 0.22-1.21) or major bleeding (HR: 1.09; 95% CI: 0.82-1.44) comparing rivaroxaban with warfarin.^2^ Furthermore, post hoc analysis of the ARISTOTLE trial included 1,236 patients with histories of cancer, of whom 157 (12.7%) had active cancer. This was the only randomized anticoagulation trial to compare efficacy and safety of apixaban (n = 76) vs warfarin (n = 81) post hoc in patients with active cancer only. The event rates were low and therefore comparison of CVA was performed, and no difference was observed in bleeding outcomes (any bleeding HR: 0.93; 95% CI: 0.55-1.56).^3^ A meta-analysis of ENGAGE AF-TIMI 48, ROCKET AF, and ARISTOTLE that included patients with histories of cancer showed similar risk for stroke (OR: 0.70; 95% CI: 0.45-1.09) and major bleeding (OR: 0.71; 95% CI: 0.31-1.64) when comparing DOACs with warfarin.^4^ Although these studies analyzed DOAC use vs warfarin in the cancer population, the majority of patients included were not clearly defined as having active cancer, in contrast to our study, which included only those with active cancer. In addition, the randomized trials had strict exclusion criteria at baseline, with less generalizability to real-world practice compared with our study.

More recently, a retrospective administrative data analysis of Medicare and claims databases in the ARISTOPHANES (Clinical and Economic Outcomes of Oral Anticoagulants in Non-Valvular Atrial Fibrillation) study of patients with NVAF and active cancer treated with DOACs or warfarin showed that apixaban was associated with a lower risk for stroke and major bleeding compared with warfarin, whereas dabigatran and rivaroxaban had similar risk for stroke and major bleeding compared with warfarin.^25^ However, we used individual medical record review to establish baseline embolic and bleeding risks, allowing propensity matching on these factors. Another recent retrospective nationwide cohort study using administrative data from the Taiwan National Health Insurance Research Database also showed that DOACs were associated with decreased risk for major adverse cardiovascular events, major adverse limb events, venous thrombosis, and major bleeding compared with warfarin in a population with NVAF and cancer.^5^ Furthermore, a recent systemic review and meta-analysis of post hoc analyses from the ROCKET AF, ENGAGE AF–TIMI 48, and ARISTOTLE trials, along with 2 additional cohort studies that compared DOACs with warfarin in patients with NVAF and cancer diagnoses, showed that DOACs had lower or similar rates of thromboembolic and bleeding events compared with patients taking warfarin.^6^ A meta-analysis of randomized controlled trials comparing DOACs with LMWH in patients with cancer did not reveal an increased risk for major bleeding; however, clinically relevant nonmajor bleeding risk was increased (risk ratio: 0.68; 95% CI: 0.54-0.86; *P* = 0.001).^11^ This clinically relevant nonmajor bleeding risk was observed mostly in patients with gastrointestinal malignancy.^11^

Similar to the aforementioned studies, our real-world, clinical chart review data show that patients with active cancer and NVAF taking DOACs had a similar risk for CVA compared with patients receiving warfarin. There was also no significant difference in overall survival between these 2 groups. In contrast to the original RE-LY (Randomized Evaluation of Long Term Anticoagulant Therapy), ROCKET AF, and ENGAGE AF–TIMI 48 trials and the meta-analysis of DOACs compared with LMWH studies, which showed the potential for DOACs to cause an increased incidence of major bleeding and/or GIB,^11^^,^^21^^,^^22^^,^^24^ our data showed that in patients with active cancer, there was no difference in GIB-free survival between patients receiving DOACs and those receiving warfarin. Additionally, we did not observe any significant difference in composite event-free (CVA, GIB, or ICH) survival or overall survival in patients with active cancer receiving DOACs compared with warfarin. We did not evaluate subgroups on the basis of type of DOAC used, because of the limited patient numbers. The majority of patients received apixaban and rivaroxaban in our propensity-matched cohort (47% and 46%, respectively) as displayed in Table 4. Therefore, it is unknown whether the findings of our study are generalizable to all DOACs compared with warfarin vs predominantly apixaban and rivaroxaban.

**Table 4:** Type of DOAC Used in Propensity-Matched and Overall Cohort

	Propensity-Matched DOAC Group (n = 195)	Overall Cohort DOAC Group (n = 842)
Apixaban	92 (47.2)	304 (36.1)
Rivaroxaban	90 (46.2)	482 (57.2)
Dabigatran	13 (6.7)	54 (6.4)
Edoxaban		3 (0.4)

Values are n (%). DOAC = direct oral anticoagulant agent.

Although current guidelines support the use of DOACs as first-line agents for anticoagulation in patients with NVAF, they do not offer specific guidance for anticoagulation in patients with NVAF and cancer.^12^^,^^26^ As a result of the paucity of data, and a lack of clear guidance in a population with heightened concern for bleeding, DOACs have been shown to be underused in patients with cancer with NVAF.^27^ It is important to acknowledge that DOACs use CYP3A4- and P-glycoprotein-mediated transport pathways, which are shared with many antineoplastic agents, which could lead to drug-drug interactions and ultimately alter the desired anticoagulant effect.^28^ However, DOACs still have substantially fewer pharmacologic and nutritional interactions than warfarin, and patients with cancer treated with warfarin are particularly vulnerable to unstable international normalized ratio control secondary to drug-drug interactions with cancer therapies, changes in dietary or nutritional status, fluctuation in renal or hepatic function, progression of their malignancies, and need for multiple procedures, ultimately predisposing them to increased risk for bleeding or thromboembolism.^29^

When compared against warfarin, DOACs offer a multitude of advantages, primarily due to quick onset of action, short half-life, low variability of serum drug levels, ease of use, no need for monitoring, and decreased concern for drug-drug interactions. These advantages could ultimately translate to increased efficacy and safety of DOACs vs warfarin in the cancer population. Although robust prospective randomized clinical trials are not available to guide anticoagulation in patients with active cancer and NVAF, our data combined with those from other recent studies, and ease of DOAC use, especially in these patients who often need interruption of anticoagulation for cancer-related procedures, reinforce that DOACs may be overall preferable to warfarin as thromboembolic prophylaxis in patients with NVAF and cancer.

### Study limitations

Our study is limited by its retrospective nature and accompanying issues of confounding, selection bias, unmeasured confounders such as additional baseline characteristics (cancer stage, cancer therapy, chronic kidney disease), and finally patient and provider anticoagulation preferences. Although we did attempt to address these limitations by performing adjudication and propensity score matching, some of the aforementioned limitations still apply. The propensity score matching resulted in a cohort with slightly higher CHA_2_DS_2_-VASC and HAS-BLED scores compared with the overall cohort, which may limit the generalizability of this study. However, the outcome event rates were similar between the overall cohort and the propensity-matched cohorts (Table 5). Principally, there was a distinct selection bias for patients who were able to tolerate anticoagulation in the setting of malignancy, whereas patients not prescribed anticoagulation secondary to their advanced comorbidities, likely related to their malignancies, would have been excluded. Furthermore, limited patient numbers impaired our ability to perform DOAC subgroup analysis. The chronological order in which anticoagulant agents were introduced into clinical practice likely introduced some bias, but this was mitigated by matching on year of anticoagulant agent initiation in the propensity score.

**Table 5:** 5-Year Outcome Event Rates Between the Overall Cohort and the Propensity-Matched Cohort

	Overall Cohort (n = 1,133)	Propensity Match (n = 390)
Death	145 (20.5)	58 (20.0)
CVA	59 (8.4)	25 (8.8)
GIB	59 (8.3)	23 (7.8)
ICH	6 (1.1)	4 (1.6)
CVA or GIB or ICH	114 (15.6)	46 (15.5)

Values are n (%). For CVA, GIB, ICH, and CVA or GIB or ICH, a Fine-Gray model was used for cumulative incidence estimates.

Abbreviations as in Tables 1 and 3.

## Conclusions

We show in this real-world data study, that DOACs were associated with similar rates of CVA, ICH, and GIB compared with warfarin in patients with active cancer and NVAF. This real-world study is one of the first to analyze patients exclusively with active cancer in whom all events were adjudicated. This reinforces what is often now adopted as standard practice in that use of DOACs is preferred over warfarin in patients with active cancer and NVAF for prevention of thromboembolic events.

Perspectives **COMPETENCY IN MEDICAL KNOWLEDGE:** In patients with NVAF and active cancer, DOACs show similar efficacy and adverse event profile compared with warfarin.

**TRANSLATIONAL OUTLOOK:** Future studies should determine the factors specific to cancer subtypes and cancer therapeutic agents that may help guide the choice of anticoagulant agent for NVAF in the setting of active cancer.

## Funding Support and Author Disclosures

The statistical analysis work is supported in part by the Cancer Center Support Grant (National Cancer Institute grant P30 CA016672). Dr Deswal is supported in part by the Ting Tsung and Wei Fong Chao Distinguished Chair. Dr Palaskas is a Cancer Prevention Research Institute of Texas Scholar and Andrew Sabin Family Foundation Fellow and is supported by Cancer Prevention Research Institute of Texas grant RP200670 and by National Institutes of Health/National Cancer Institute grant 1P01CA261669-01; has received consulting fees from Replimmune and Kiniksa; and has received an honorarium from Patient Education Resource. All other authors have reported that they have no relationships relevant to the contents of this paper to disclose to disclose.
