# Supplementary material for: Outcomes by Class of Anticoagulant Use for Nonvalvular Atrial Fibrillation in Patients With Active Cancer
Source: JACC CardioOncol. 2022 Sep 20;4(3):341–50. doi: 10.1016/j.jaccao.2022.07.004 (PMC9537073; doi:10.1016/j.jaccao.2022.07.004)
Supplement: Supplemental Tables 1–5 [file mmc1.docx]

**Supplemental Material**

**Outcomes of Stroke and Bleeding by Class of Anticoagulant Use For Non-valvular Atrial Fibrillation In Patients With Active Cancer**

Adam S. Potter MD, PhD^a^, Ashley Patel MD^b^, Muzamil Khawaja MD^b^, Christopher Chen MD^b^, Henry Zheng MD^b^, Jessica Kaczmarek MD^b^, Feng Gao MD^b^, Kaveh Karimzad MD^a^, Juhee Song PhD^c^, Efstratios Koutroumpakis MD^a^, Shaden Khalaf MD^a^, Cezar Iliescu MD^a^, Anita Deswal MD, MPH^a^, Nicolas L. Palaskas MD^a^

^a^Department of Cardiology, University of Texas MD Anderson Cancer Center, Houston, TX

^b^Department of Internal Medicine, Baylor College of Medicine, Houston, TX Houston, TX

^c^Department of Biostatistics, University of Texas MD Anderson Cancer Center, Houston, TX

**Supplemental Table 1.** Multivariable logistic regression model in predicting the receipt of Warfarin

| **Covariate** | **OR (95% CI)** | **P-value** |
| --- | --- | --- |
|  |  |  |
| Sex- Female | 0.790 (0.553-1.128) | 0.20 |
| Age, years (In 1 unit change) | 0.975 (0.954-0.997) | 0.025 |
| HAS-BLED Score (In 1 unit change) | 3.102 (2.220-4.334) | <0.001 |
| Year Anticoagulation Started |  |  |
| 1995-2010 | 1.000 |  |
| 2011-2015 | 0.231 (0.134-0.398) | <0.001 |
| 2016-2020 | 0.031 (0.018-0.056) | <0.001 |
| Heart Failure | 1.955 (1.277-2.991) | 0.002 |
| Hypertension | 1.722 (1.089-2.723) | 0.020 |
| Uncontrolled Hypertension | 0.213 (0.112-0.404) | <0.001 |
| Diabetes | 0.966 (0.641-1.453) | 0.87 |
| CVA | 0.311 (0.169-0.573) | <0.001 |
| Vascular Disease | 1.389 (0.892-2.164) | 0.15 |
| Prior Major Bleed | 0.236 (0.108-0.517) | <0.001 |
| Alcohol Use | 0.320 (0.106-0.970) | 0.044 |
| Renal disease | 0.401 (0.180-0.896) | 0.026 |
| Medication Predisposing to Bleed | 0.190 (0.109-0.333) | <0.001 |
| Gastrointestinal Malignancy | 1.080 (0.648-1.799) | 0.77 |

**Supplemental Table 2.** Cancer types in “Other” category of overall cohort by anticoagulant

| **Cancer type** | **NOAC**  **(n=126)** | **Warfarin**  **(n=37)** | **p-value** |
| --- | --- | --- | --- |
| **Other category in overall cohort, n (%)** |  |  | 0.99 |
| Head and Neck Malignancy | 70 (56) | 20 (54) |  |
| Gynecologic Malignancy | 29 (23) | 9 (24) |  |
| Sarcoma | 15 (12) | 5 (14) |  |
| Central Nervous System Malignancy | 10 (8) | 3 (8) |  |
| Bone Malignancy | 2 (2) | 0 (0) |  |

**Supplemental Table 3**- Differences in baseline characteristics by sex of overall cohort

| **Characteristic** | **Male** | **Female** | **P-value** |
| --- | --- | --- | --- |
|  | (n = 662) | (n = 471) |  |
| Type of Anticoagulant – n (%) |  |  |  |
| NOAC | 473 (71%) | 369 (78%) | 0.009 |
| Warfarin | 189 (29%) | 102 (22%) |  |
| Age, years (Mean ± SD) | 72.4 ± 8.8 | 72.6 ± 8.8 | 0.62 |
| Race – n (%) |  |  | 0.26 |
| Black | 33 (5) | 37 (8) |  |
| White | 593 (90) | 409 (87) |  |
| Other | 36 (5) | 25 (5) |  |
| CHA2DS2-VASc Score (Mean ± SD) | 3.0 ± 1.7 | 3.8 ± 1.7 | <0.001 |
| HAS-BLED Score (Mean ± SD) | 1.9 ± 1.1 | 1.8 ± 1.0 | 0.006 |
| Year Anticoagulation Started, n (%) |  |  | 0.005 |
| 1995-2010 | 74 (11) | 52 (11) |  |
| 2011-2015 | 216 (33) | 113 (24) |  |
| 2016-2020 | 372 (56) | 306 (65) |  |
| Comorbidities – n (%) |  |  |  |
| Heart Failure | 118 (18) | 72 (15) | 0.26 |
| Hypertension | 533 (81) | 362 (77) | 0.14 |
| Uncontrolled Hypertension | 97 (15) | 60 (13) | 0.36 |
| Diabetes | 160 (24) | 102 (22) | 0.32 |
| CVA | 108 (16) | 64 (14) | 0.21 |
| Vascular Disease | 222 (34) | 104 (22) | <0.001 |
| Prior Major Bleed | 47 (7) | 36 (8) | 0.73 |
| Hyperlipidemia | 569 (86) | 390 (83) | 0.15 |
| Alcohol Use | 26 (4) | 5 (1) | 0.004 |
| Renal Disease | 52 (8) | 10 (2) | <0.001 |
| Labile INR | 74 (11) | 36 (8) | 0.048 |
| Liver Disease | 19 (3) | 8 (2) | 0.20 |
| Medication Predisposing to Bleed | 228 (34) | 163 (35) | 0.95 |
| Cancer Type |  |  | <0.001 |
| Breast | 2 (0.3) | 170 (36) |  |
| Genitourinary | 200 (30) | 21 (4) |  |
| Gastrointestinal | 87 (13) | 50 (11) |  |
| Liquid^*^ | 154 (23) | 82 (17) |  |
| Lung | 55 (8) | 37 (8) |  |
| Skin^**^ | 82 (12) | 30 (6) |  |
| Other^***^ | 82 (12) | 81 (17) |  |

^*Liquid = AML, ALL, CLL, CML, DLBCL, MM, MF, MGUS, PV, ET, Follicular Lymphoma, Hairy Cell, HL, Marginal Zone Lymphoma^

^**Skin = SCC, Melanoma, BCC, mycosis fungoides, Dermatofibroma, Merkel Cell^

^***Other = Sarcoma, Gynecologic, CNS, Bone, Head and Neck^

**Supplemental Table 4.** General characteristics of matched versus unmatched cohorts.

| **Characteristic** | **Unmatched**  **n=1,133** | **Matched**  **n=390** |
| --- | --- | --- |
| Sex- n (%) |  |  |
| Male | 662 (58) | 239 (61) |
| Female | 471 (42) | 151 (39) |
| Age, years (Mean ± SD) | 72.9 ± 8.8 | 72.0 ± 8.8 |
| Race – n (%) |  |  |
| Black | 70 (6) | 21 (5) |
| White | 1002 (88) | 352 (90) |
| Other | 61 (5) | 17 (4) |
| CHA_2_DS_2_-VASc Score (Mean ± SD) | 3.3 ± 1.7 | 3.5 ± 1.7 |
| HAS-BLED Score (Mean ± SD) | 1.9 ± 1.0 | 1.8 ± 1.0 |
| Year Anticoagulation Started, n (%) |  |  |
| 1995-2010 | 126 (11) | 52 (13) |
| 2011-2015 | 329 (29) | 228 (58) |
| 2016-2020 | 678 (60) | 110 (28) |
| Comorbidities – n (%) |  |  |
| Heart Failure | 190 (17) | 88 (23) |
| Hypertension | 895 (79) | 326 (84) |
| Uncontrolled Hypertension | 157 (14) | 46 (12) |
| Diabetes | 262 (23) | 99 (25) |
| CVA | 172 (15) | 66 (17) |
| Vascular Disease | 326 (29) | 123 (32) |
| Prior Major Bleed | 83 (7) | 28 (7) |
| Hyperlipidemia | 959 (85) | 327 (84) |
| Alcohol Use | 31 (3) | 7 (2) |
| Renal Disease | 62 (5) | 24 (6) |
| Labile INR | 110 (10) | 45 (12) |
| Liver Disease | 27 (2) | 10 (3) |
| Medication Predisposing to Bleed | 391 (35) | 98 (25) |
| Cancer Type |  |  |
| Breast | 172 (15) | 57 (15) |
| Genitourinary | 221 (20) | 86 (22) |
| Gastrointestinal | 137 (12) | 53 (14) |
| Liquid^*^ | 236 (21) | 72 (18) |
| Lung | 92 (8) | 29 (7) |
| Skin^**^ | 112 (10) | 42 (11) |
| Other^***^ | 163 (14) | 51 (13) |

^*Liquid = AML, ALL, CLL, CML, DLBCL, MM, MF, MGUS, PV, ET, Follicular Lymphoma, Hairy Cell, HL, Marginal Zone Lymphoma^

^**Skin = SCC, Melanoma, BCC, mycosis fungoides, Dermatofibroma, Merkel Cell^

^***Other = Sarcoma, Gynecologic, CNS, Bone, Head and Neck^

**Supplemental Table 5**. Cox Proportional Hazards Models for CVA, ICH, GIB, Composite Event using 1:1 propensity score matched cohorts

|  | **Cox Proportional Hazards Models** | | | | | | | |
| --- | --- | --- | --- | --- | --- | --- | --- | --- |
|  | **CVA** | | **ICH** | | **GIB** | | **Composite Event** | |
| **Covariate** | **aHR**  **(95% CI)** | **p-value** | **aHR**  **(95% CI)** | **p-value** | **aHR**  **(95% CI)** | **p-value** | **aHR**  **(95% CI)** | **p-value** |
| **Type of Anticoagulant** |  |  |  |  |  |  |  |  |
| NOAC | 1.000 | - | 1.000 |  | 1.000 |  | 1.000 |  |
| Warfarin | 0.761  (0.345-1.677) | 0.50 | 0.761  (0.345-1.677) | 0.50 | 1.881  (0.798-4.439) | 0.15 | 1.194  (0.668-2.134) | 0.55 |

(aHR= adjusted hazard ratio, NOAC = Non-Vitamin K Antagonist Oral Anticoagulant, CVA = Cerebrovascular Accident, ICH = Intracranial Hemorrhage, GIB = Gastrointestinal Bleed, Composite Event = CVA, ICH, and/or GIB).
